# Loci associated with conception rate in crossbred beef heifers

**DOI:** 10.1371/journal.pone.0230422

**Published:** 2020-04-09

**Authors:** K. F. Oliver, T. W. Geary, J. N. Kiser, J. M. Galliou, M. L. Van Emon, C. M. Seabury, T. E. Spencer, H. L. Neibergs

**Affiliations:** 1 Department of Animal Sciences, Washington State University, Pullman, WA, United States of America; 2 USDA-ARS Fort Keogh LARRL, Miles City, MT, United States of America; 3 Department of Animal and Range Sciences, Montana State University, Bozeman, MT, United States of America; 4 Department of Veterinary Pathobiology, College of Veterinary Medicine, Texas A&M University, TX, United States of America; 5 Division of Animal Sciences, University of Missouri, Columbia, MO, United States of America; University of Florida, UNITED STATES

## Abstract

The inability of beef cattle to maintain full term pregnancies has become an economic concern for the beef industry. Herd management and nutritional improvements have alleviated environmental impacts on embryonic and fetal loss, yet additional gains can be made through genomic selection. The objectives of this study were to identify loci and gene-sets in crossbred beef heifers associated with the number of services required to become pregnant (TBRD) and heifer conception rate at first service (HCR1). Heifers (*n =* 709) from a commercial beef operation underwent one round of artificial insemination, before exposure to bulls for natural service for 50 days. Pregnancy and time of conception was determined by ultrasound 35 days after the breeding season. Heifers were genotyped using the GeneSeek (Lincoln, NE) Bovine GGP50K BeadChip prior to genome-wide association analyses (GWAA) conducted using an EIGENSTRAT-like model to identify loci associated (*P* < 1 × 10^−5^) with TBRD and HCR1. One locus was associated (*P* = 8.97 × 10^−6^) with TBRD on BTA19 and included the positional candidate gene *ASIC2*, which is differentially expressed in the endometrium of fertility classified heifers, and the positional candidate gene, *SPACA3*. Gene-set enrichment analyses using SNP (GSEA-SNP) data, was performed and identified one gene-set, oxidoreductase activity, acting on paired donors, with incorporation or reduction of molecular oxygen as enriched (*NES* = 3.15) with TBRD and contained nine leading edge genes that contributed to the enrichment of the gene set. The enriched gene-set is involved in catalyzing oxidation-reduction reactions, which have been associated with oxidative stressors impacting pregnancy success. No loci were associated nor gene-sets enriched with HCR1. Identification of loci, positional candidate genes, gene-sets and leading edge genes enriched for fertility facilitate genomic selection that allows producers to select for reproductively superior cattle, reduce costs associated with infertility, and increase percent calf crop.

## Introduction

Reproductive efficiency is a critical component for the longevity of beef cattle production [1]. Even though fertilization rates are over 90% [2,3] previous findings estimated that only 60% of fertilized oocytes reached full term [4,5]. Failure of cows and heifers to maintain pregnancy is a substantial loss to producers and accounts for most of the reproductive costs incurred [6]. By reducing infertility by 5%, the beef industry would save over $90 million annually [7,8].

In the beef industry, the use of fertility measures for EPD is limited to traits with open-ended breeding season lengths [9,10]. Using traditional EPD for selection of complex traits, such as fertility, is difficult as they often display lower heritability and prolonged or late-in life trait expression [11,12]. Genomic selection improves selection accuracy and therefore is particularly useful in complex traits with lower heritability or traits that occur late-in life. Genome-wide association analysis (GWAA) and gene-set enrichment analysis using SNP (GSEA-SNP) data are often used to identify associations of genetic variants with traits for genomic selection [11,13]. Thus, the objectives of this study were to identify loci, gene-sets and leading edge genes associated with the number of services required to become pregnant (TBRD) and heifer conception rate at first service (HCR1) in commercial crossbred beef heifers.

## Materials and methods

The protocol for this study was in accordance with the USDA-ARS Fort Keogh Animal Care and Use Committee (USDA-ARS approval No. 040418–1) and a memo of understanding with Washington State University’s Animal Care and Use Committee.

### Study population

The study population consisted of 709 Angus-Salers heifers from a commercial beef cattle ranch located in Montana. Breed percentages for this herd were approximately one-quarter to three-eighths Salers and three-fourths to five-eighths Angus. Heifers were approximately 14 months of age and weighed approximately 350 kg (~60% of mature body weight) at time of first service. Estrus was synchronized in heifers with melengestrol acetate (0.5 mg MGA/hd/d in feed; Pfizer, New York, NY) for 14 days, and an injection of prostaglandin (5 ml Lutalyse; Pfizer, New York, NY) administered 19 days after the last day of MGA feeding. At observed estrus, heifers were bred by AI (day 0) to the same Angus bull. The heifers were then split between two pastures located in Hobson, MT (*n* = 388) and Melstone, MT (*n* = 321) with two and three year old bulls (added 10 days after AI) for 50 days with a bull to heifer ratio of 1:40. A t-test determined pasture location was not significant for TBRD (*P* = 0.28) or HCR1 (*P* = 0.25). Ultrasound was used to determine pregnancy and age embryos approximately 35 days after the conclusion of the breeding season.

Prior to genotyping, heifers were removed if they had any health issues or discrepancies with identification tags. Of the remaining 676 heifers, 300 were selected for genotyping and included 95 that conceived from the first service (AI), 94 that conceived from the second service (approximately day 20), 50 that conceived from the third service (approximately day 40), and 61 heifers that never conceived. Due to the limited number of heifers that conceived from the third service and those that never conceived, all individuals for these groups were genotyped, whereas the heifers from the first and second services were chosen randomly for genotyping.

### Phenotypes

The TBRD analysis compared 95 heifers pregnant to the first service, 94 heifers pregnant to the second service and 50 pregnant to the third service. The 61 heifers that never conceived were excluded from this analysis. The HCR1 analysis compared 95 heifers that were pregnant to the first service to 205 heifers that did not conceive to the first service, which included heifers that did not conceive during the breeding season. Understanding how TBRD and HCR1 are associated with loci regulating pregnancy will provide a better understanding of the complex mechanisms involved in pregnancy success.

### DNA extraction and genotyping

Whole blood (~8 ml) was collected via tail venipuncture into EDTA tubes. White blood cells were isolated and DNA extracted using the Puregene DNA extraction kit (Gentra, Minneapolis, MN) as per manufacturer’s instructions. DNA was quantified and quality assessed using the Nanodrop 1000 spectrophotometer (Thermo Fisher Scientific, Wilmington, DE). Samples were genotyped using the GeneSeek Bovine GGP50K BeadChip (Lincoln, NE). The GGP50 BeadChip contains 47,843 SNP with a SNP genotype occurring an average of every 59 kb [14].

### Quality control

Quality control filtering removed SNP with a call rate of < 90% (1,542 SNP removed), a minor allele frequency < 0.01% (1,859 SNP removed), and SNP that failed Hardy-Weinberg equilibrium (*P* < 1 × 10^−50^, 4 SNP removed), leaving 44,419 SNP for the analysis. Quality control filtering for heifers removed individuals with less than 90% of their genotypes called (10 heifers removed), duplicate animals (2 heifers removed), and heifers with genetic and anatomical sex discrepancies (3 heifers removed). This resulted in 228 heifers remaining for TBRD (89 pregnant to the first service, 93 pregnant to the second service, and 46 pregnant to the third service) and 285 heifers remaining for HCR1 (the same heifers as in TBRD with the addition of 57 heifers that did not conceive).

### Genome-wide association analysis

A GWAA was conducted for TBRD and HCR1 using an approach similar to EIGENSTRAT [15], that corrects for population stratification using principal components analysis (PCA), in the SNP and Variation Suite (SVS) software v 8.1 [16]. The first 10 principal components were used for the analyses. After PCA correction, a linear regression analysis using an additive model was conducted in SVS. The genomic inflation factors (λ_GC_) were near or at one (λ_GC_ = 1.02 for TBRD and λ_GC_ = 1.00 for HCR1).

The significance threshold for an association was based on the Wellcome Trust Case Control Consortium recommendation for unadjusted *P* values, where *P* values between 1 × 10^−5^ and 5 × 10^−7^ indicated a moderate association and unadjusted *P* < 1 × 10^−7^ provided evidence for a strong association [17]. Positional candidate genes were identified within a 28.4 kb region (14.2 kb upstream and 14.2 kb downstream) surrounding the associated SNP based on the average haplotype block size of crossbred beef cattle using the method previously described by Gabriel and coworkers (2002) [18].

### Gene-set enrichment analysis-single nucleotide polymorphism

The GSEA-SNP analysis was computed with the GenGen software package (Holden et al., 2008). SNP were mapped to corresponding genes based on the UMD 3.1 assembly (ftp://ftp.cbcb.umd.edu/pub/data/Bos_taurus/). The most significant SNP located within a gene or within a haplotype block size of 14.2 kb was used for the gene proxy for 19,723 genes as described previously by Neupane and coworkers (2017) [19]. Briefly, the SNP used as gene proxies were ranked and ordered by their significance and then enrichment scores (ES) were computed based on the presence or absence of each gene in the gene-set present. The ES statistics were calculated similarly to the weighted Kolmogorov-Smirnov-like statistic [20]. Gene-sets were taken from Gene Ontology (GO), Reactome, Kyoto Encyclopedia of Genes and Genomes (KEGG), Biocarta, and Protein Analysis Through Evolutionary Relationships (PANTHER). Genes that positively contributed to the peak ES of the gene-set, are leading edge genes and were identified as associated with either TBRD or HCR1. The permuted *P* value for each gene set was calculated using GenABEL in R with 10,000 phenotype-based permutations [21]. Enrichment scores were normalized (normalized enrichment scores; *NES*) to account for variances in the number of genes for each gene-set. For this study, a gene-set was considered enriched if the *NES* > 3.0.

## Results

### Genome-wide association analysis

**Fig 1:**
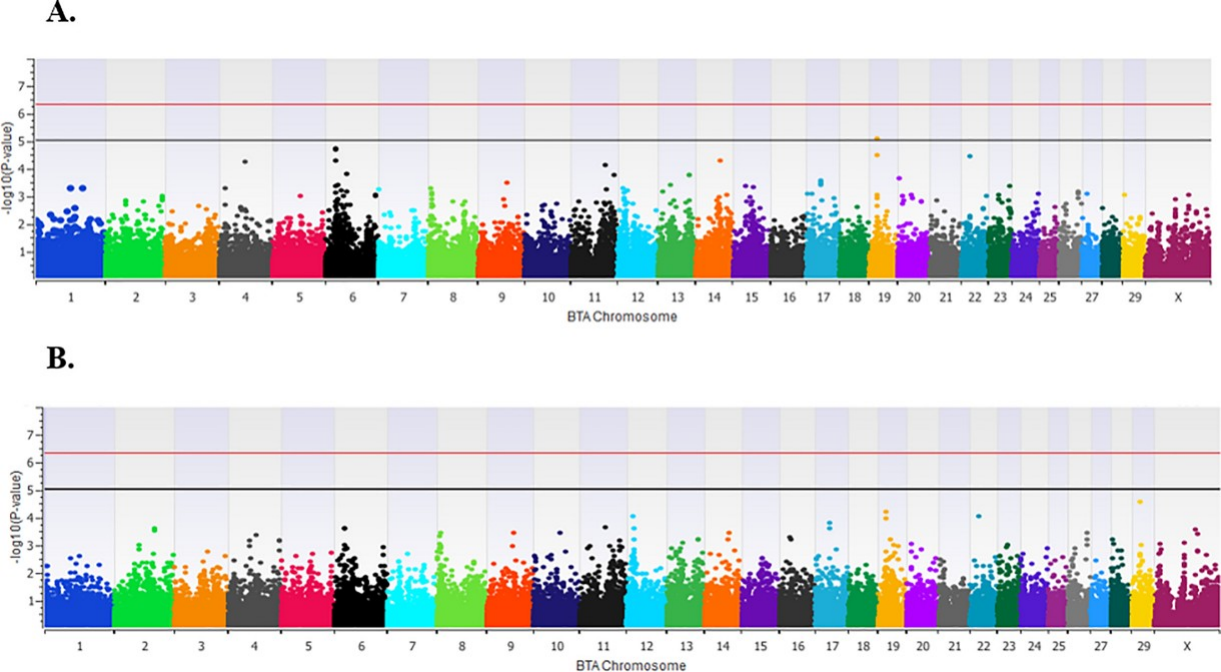
Manhattan plots of loci associated with fertility in a genome-wide association analysis using an approach similar to EIGENSTRAT. Panel A identified the locus associated with the number of services required to successfully conceive and maintain pregnancy (TBRD), and heifer conception rate at first service (HCR1) was identified in Panel B. SNP located between the black and red lines provide evidence of a moderate association (*P* < 1 × 10^−5^) with TBRD.

One locus was moderately associated (*P* = 8.97 × 10^−6^) with TBRD on BTA19 (Fig 1A; Table 1). Two positional candidate genes were associated with this locus. The associated SNP (*rs110962436*) was located 626 bp downstream of the acid sensing ion channel subunit 2 (*ASIC2)* gene and approximately 10 kb from the sperm acrosome associated 3 (*SPACA3*) gene. No loci were associated with HCR1 (Fig 1B), although two loci trended toward an association, one on BTA29 (*rs134906513*, *P* = 3.01 × 10^−5^) and one on BTA19 (*rs110962436*, *P* = 6.87 × 10^−5^), which was the locus identified as associated with TBRD.

**Table 1:** Loci, gene-sets, and leading edge genes associated with the number of services required to successfully conceive and maintain pregnancy (TBRD) for 300 crossbred Angus/Salers heifers.

Association Test	BTA /Gene Set^1^	Significance^2^	Positional Candidate Genes/Leading Edge Genes^3^
*GWAA*	19(17)	8.97 × 10^−06^	*ASIC2*, *SPACA3*
*GSEA-SNP*	GO: 0016705	*NES* = 3.15	*MICAL2*, *PAH*, *PTGS1*, *CYP2D14*, *PHYH*, *DBH*, *P4HA3*, *CYP19A1*, *HMOX1*

^1^*Bos taurus* (BTA) chromosome location of the loci associated with fertility followed by the location of the associated SNP in megabases (Mb) in parentheses for GWAA; for GSEA-SNP the enriched gene set was from Gene Ontology (GO).

^2^The significance (*P* < 1 × 10^−5^) was taken from the Wellcome Trust Case Control Consortium, 2007 for uncorrected *P* values for the GWAA; for the GSEA-SNP significance was determined by a normalized enrichment score (*NES >3*.*0*)

^3^Positional candidate genes were defined as genes located within 28.4 kb of the associated SNP(s) and leading edge genes were the genes positively influencing the enrichment scores for the gene set. Gene locations were based on UMD 3.1.

### Gene-set enrichment analysis-single nucleotide polymorphism

The GSEA-SNP identified one gene-set (oxidoreductase activity acting on paired donors with incorporation or reduction of molecular oxygen, GO: 0016705) that was enriched (*NES* = 3.15) for TBRD. This gene set included 34 genes with nine leading edge genes associated with TBRD (Table 1). No gene-sets were enriched for HCR1.

## Discussion

That there were no loci associated with HCR1 may have been confounded by the pubertal status of the heifers prior to administration of MGA. As heifers were not tested to identify if they were cycling prior to administration of MGA, it is possible that their first estrus was at the time of breeding. Heifers bred to their first estrus or less likely to conceive than heifers who have had a previous estrous cycle [22]. If heifers had all been bred after at least one estrous cycle, it is possible that an association with HCR1 would have been identified.

The locus associated with TBRD was near *ASIC2*. Mutations of *ASIC2* lead to cellular death and cause changes to the inactivation process of pH dependent channels [23,24]. In the oviduct, expression of *ASIC2* is localized to the ciliated cells when an acidic state occurs [25]. Acidic environments usually indicate an abnormal condition such as inflammation [25]. Inflammation of the female reproductive tract is negatively correlated with pregnancy success. Differential expression of *ASIC2* occurred in the endometrium between fertility classified heifers, supporting the idea that *ASIC2* has functions that may be associated with pregnancy success [26].

The second positional candidate gene associated with TBRD is *SPACA3*, which encodes sperm protein reactive with anti-sperm antibody (SPRASA) involved in sperm adhesion to the egg, fertilization, and embryonic development in females [27]. Inhibition of SPRASA antiserum interrupted embryonic development at the morula stage [27], which would be detrimental in the successful establishment and maintenance of pregnancy in heifers.

The gene-set enriched for TBRD, oxidoreductase activity acting on paired donors with incorporation or reduction of molecular oxygen (GO: 0016705), is involved in catalysis of oxidation-reduction reactions [28, 29], which can contribute to oxidative stressed environments. Pregnancy is a state of oxidative stress [30] as placental mitochondria have increased metabolic activity and reduced scavenging power of antioxidants [31]. In humans, early pregnancy oxidative stress is due to the intrauterine environment being low in oxygen [32], placing the placenta and fetus in a hypoxic environment [31]. This hypoxic environment is crucial in controlling oxygen homeostasis and is an essential factor for early embryonic and placental development [33] in sheep [34], humans [35], and mice [36]. Additionally, reactive oxygen species have key roles in apoptosis during early embryonic development [37, 38], maintaining homeostasis within the uterine endometrium for implantation [39–43], and may be initiated either intrinsically by the mitochondrial pathway or extrinsically through membrane-associated death receptors [38]. Adverse pregnancy outcomes associated with increased levels of reactive oxygen species have been linked with reproductive inefficiencies such as spontaneous abortion, intrauterine growth restriction and unexplained infertility [38, 44–45].

The nine leading edge genes (Table 1) in GO: 0016705 had various roles ranging from cell proliferation (*MICAL2)* [46] to encoding enzymes for proper peroxisome function (*PYPH*) [47]. Four leading edge genes influence the ovarian (*DBH*, *MICAL2*, *CYP19A1)* and uterine environments (*PTGS1)*, while one (*HMOX1*) is involved in the immune response (Table 1). The leading edge gene *DBH* is thought to be associated with oxidative stress in the follicle [48–51] while *HMOX1* encodes for HO-1, a unique regulator involved in prevention of tissue injury and modulates both innate and adaptive immune response [52, 53]. Three (*PHYH*, *MICAL2*, *HMOX1*) of the nine leading edge genes are differentially expressed in the endometrium (*MICAL2*, *HMOX1*) and conceptus (*PHYH*) of fertility classified crossbred beef heifers [26]. This supports the case that these genes have underlying influences on pregnancy establishment within the uterine environment.

## Conclusion

This study improves understanding of the loci associated with fertility in crossbred beef heifers and the genes that may play a role in successful establishment of pregnancy. One positional candidate gene (*ASIC2*) and three of the leading edge genes (*PHYH*, *MICAL2*, *HMOX1*) identified are supported in their fertility role by their differential expression in fertility-classified heifers. Similarly, the positional candidate genes, *ASIC2* and *SPACA3*, associated with TBRD, have functional relevance to the establishment of a successful pregnancy in the first weeks of gestation. These results provide preliminary information that may enhance use of genomic selection in the beef industry to better select for reproductively superior heifers and improve our understanding of factors contributing to embryonic losses.
